# Multidimensional health status and its impact on health care consumption behavior among elderly people with chronic diseases: evidence from CHARLS in China

**DOI:** 10.3389/fpsyg.2025.1543982

**Published:** 2025-04-16

**Authors:** Qi Wang, Yuanyuan Zhang, Xin Miao, Jinao Chen, Linlin Zhang

**Affiliations:** ^1^School of Medical and Health Engineering, Changzhou University, Changzhou, Jiangsu, China; ^2^Department of Nursing, Changzhou University, Changzhou, Jiangsu, China; ^3^Nanjing Lishui District Hospital of Traditional Chinese Medicine, Nanjing, Jiangsu, China; ^4^School of Physical Education, Changzhou University, Changzhou, Jiangsu, China

**Keywords:** elderly, chronic diseases, multidimensional health, health care consumption behavior, CHARLS

## Abstract

**Purpose:**

This study aims to provide a comprehensive understanding of the multidimensional health status of elderly individuals with chronic diseases in China and examine its impact on their healthcare consumption behavior.

**Methods:**

A chi-square test was conducted to assess the variations in healthcare consumption behavior across different multidimensional health categories. Additionally, a logistic regression model was employed to identify the key determinants influencing healthcare consumption among elderly individuals with chronic diseases from a multidimensional health perspective.

**Results:**

The chi-square test results indicated a statistically significant association between multidimensional health and healthcare consumption behavior (*p* < 0.05). Furthermore, the logistic regression analysis identified dyslipidemia, regular participation in social activities, and children’s financial support (≥¥5,801 yuan per year) as significant contributors to healthcare consumption behavior among elderly individuals with chronic conditions (*p* < 0.05).

**Conclusion:**

The findings of this study suggest that both physical and social health play a positive role in enhancing healthcare consumption behavior among elderly individuals with chronic diseases (*p* < 0.05). These results highlight the importance of targeted policy interventions that integrate physical health management and social engagement strategies to improve healthcare accessibility and utilization in this population.

## 1 Introduction

With the continuous improvement in living standards, public awareness of health has deepened, and healthcare has gradually become a national strategic priority. Health plays a crucial role at the individual, societal, and national levels (Wallace et al., 2021). In this context, countries such as the United States (Abdalla et al., 2023), the United Kingdom (Viberg et al., 2023), and Canada (Jean-Philippe et al., 2022) have implemented comprehensive health strategies aimed at enhancing public health and mitigating health risks. In recent years, with rapid socio-economic development and an accelerating aging population, China has also placed increasing emphasis on public health. Since the enforcement of the strict “family planning” policy in the late 1970s, China’s fertility rate has steadily declined, leading to a demographic shift toward an aging society. The proportion of individuals aged 65 and above has risen from 6.96% in 2010 to 14.2% in 2021, officially classifying China as a deeply aging society (Yu et al., 2023).

Chronic diseases in this study refer to long-term, non-communicable diseases (NCDs) that require ongoing management and are difficult to cure. According to the World Health Organization (WHO), chronic diseases significantly impact physical, mental, and social well-being, increasing the healthcare burden, especially among older adults. Based on CHARLS 2020 data, this study includes 15 common chronic diseases, such as hypertension, diabetes, heart disease, arthritis, and digestive diseases. The prevalence of these conditions increases with age, affecting multiple health dimensions and influencing healthcare consumption behavior (Xu et al., 2023). With age, the function of various organs of the body of the elderly gradually decline, and in the long-term life may have formed some bad habits, the prevalence of chronic diseases in the elderly population is as high as 71.36% (Lu et al., 1982). According to the World Health Organization, about 41 million people die of chronic diseases every year, accounting for 70% of all deaths worldwide (Chen et al., 2023). In China, with the aggravation of population aging and changes in lifestyles, the number of patients with chronic diseases continues to increase, and the proportion of people aged 60 years and over suffering from one or more chronic diseases is as high as 35.1 per cent (Hu et al., 2024). Patients with chronic diseases often require long-term treatment and care, which will not only impose a heavy financial burden on their families, but also affect their work and quality of life. In addition, chronic diseases can increase the psychological pressure on patients, leading to the occurrence of psychological problems such as anxiety and depression (Li et al., 2023). The arrival of the “silver age” has made the aging situation in China increasingly serious, and the response to aging requires not only consideration of the population pressure in terms of quantity and scale, but also observation of the ensuing population health problems. The increasing burden of chronic diseases among aging populations presents a significant public health challenge, particularly in rapidly aging societies such as China. In response to these challenges, China’s Medium-and Long-Term Plan for the Prevention and Treatment of Chronic Diseases (2017–2025), released by the General Office of the State Council of China, emphasizes a shift from disease treatment to health management, advocating for strengthened health education to promote healthier behaviors (Yan and Mi, 2021). Similarly, the World Health Organization (WHO) defines health as a state of physical, mental, and social well-being, rather than merely the absence of disease or frailty. Therefore, exploring elderly health should adopt a multidimensional approach that captures the complex interplay between different health determinants (Wang et al., 2020). This study adopts a multidimensional health perspective, aligning with WHO’s framework, which considers four key dimensions: (1) Self-Rated Health (SRH), representing an individual’s subjective evaluation of overall health; (2) Physical Health, assessed through activities of daily living (ADL, IADL) and the presence of chronic diseases; (3) Mental Health, measured using the CES-D10 depression scale and life satisfaction; and (4) Social Health, which encompasses social participation and financial and emotional support from children. While prior research has mainly focused on the direct effects of individual health dimensions on healthcare utilization, fewer studies have examined their combined and interactive effects on medical consumption behavior. Given that elderly individuals often experience multiple health challenges simultaneously, this study aims to fill this gap by analyzing how multidimensional health factors, including their interaction effects, influence healthcare consumption behaviors. By incorporating these perspectives, this research provides critical insights into elderly healthcare decision-making, offering policy recommendations for improving healthcare accessibility, financial support programs, and social engagement initiatives in an aging society (Zhang et al., 2024). SRH is recognized as an important indicator in health studies of older populations, and improving or maintaining SRH is associated with a variety of positive health factors (Almevall et al., 2024). A study of older patients with type 2 diabetes said poorer self-rated health was associated with faster cognitive decline and more severe small vessel disease (Ramsingh et al., 2024). Physical health includes normal development of all parts of the body, good physical fitness, normal physiological indicators, etc. (Harris et al., 2024). Mental health gets attention in the digital age (Lee et al., 2024; Tuan et al., 2024), multiple studies have shown significant correlations between discussion boards on social platforms for stress and emotion and levels of mental stress, informing the development of mental health interventions. Social health is a comprehensive concept that encompasses a wide range of social relationships, from the individual level to broader socio-cultural factors, which play a crucial role in the health of individuals, especially older people (Stafford et al., 2024).

With the continuous development of the economy and the increasing improvement of living standards, the health care industry is developing rapidly and has a bright future. Health-care consumption behavior, as a kind of active maintenance or promotion of health, plays an increasingly important role in the rehabilitation of chronic diseases (Hensher et al., 2024).

## 2 Methods

### 2.1 Data

The data used in this study come from the China Health and Retirement Longitudinal Study (CHARLS), a large-scale interdisciplinary survey program conducted by the National Development Research Institute of Peking University. CHARLS is a longitudinal, household-based survey, with a national baseline survey initiated in 2011 and tracked every 2–3 years. The investigation was approved by the Biomedical Ethics Committee of Peking University (IRB00001052-11015). By the time the national follow-up is completed in 2020, the sample size will cover 27 provinces (autonomous regions and municipalities directly under the central government), 125 counties, 446 communities (villages), 11,400 households, and a sample of 19,300 middle-aged and elderly people aged 45 and over, with a nationally representative selection of samples, which will provide a reliable source of data for this study.

### 2.2 Measures

#### 2.2.1 General demographic characteristics

Due to the high mobility of China’s urban and rural populations, relying on the location of the household registration to classify the target group does not more accurately reflect the social attributes of the individual. In this study, the current place of residence was chosen as the main reference for the classification of urban–rural attributes, an indicator that more accurately reflects the possible influence of medical conditions and living standards in the respondent’s region on the respondent’s health care consumption behavior (Ruan, 2023). Therefore, the general demographic characteristics of this study include 10 independent variables of gender, marriage, age, education, type of residence, whether they currently drink alcohol, whether they currently smoke, whether they are retired, whether they have health insurance, and whether they have pension insurance.

#### 2.2.2 Dependent variable

The dependent variable of this study is health care consumption behavior. It was based on the specific question in the CHARLS 2020 questionnaire, i.e., “Expenditures on health care costs (including fitness and exercise and product equipment, health care products) in the past year?” to obtain. In this study, participants who spent “0¥” on health care consumption were coded as “0” for no health care consumption behavior, otherwise they were coded as “1” for health care consumption behavior.

#### 2.2.3 Independent variable

The independent variable of this study was multidimensional health status, which was set according to the definition of health with 4 dimensions of SRH, physical health, mental health, and social health (Grzywacz and Keyes, 2004).

##### 2.2.3.1 Self-rated health dimension

Self-rated health was obtained through the CHARLS 2020 questionnaire “How do you think your health is?.” The option “do not know” was treated as a vacant value; for the purpose of interpretation, the rest of the options were reverse scored in this study, i.e., the scores were assigned from “1 very good” to “5 very bad” in the order of “5, 4, 3, 2, 1.” The higher the total score, the better the health status.

##### 2.2.3.2 Physical health dimension

The physical health dimension was operationalized through physical activities ability (Zheng et al., 2022) and chronic disease (Fan, 2022).

###### 2.2.3.2.1 Physical activities ability

Physical activities ability in this study relied on the Activity of Daily Living (ADL) and Instrument activity of Daily Living (IADL) recorded in the CHARLS 2020 questionnaire (Zheng et al., 2022). These three independent variables were converted to binary data by the following method (Zheng et al., 2022). The CHARLS 2020 questionnaire includes six entries from the ADL scale (eating, bathing, dressing, bowel control, toileting, and bed and chair transfers) and six entries from the IADL scale (telephone use, shopping, food preparation, housework, medication use, and financial management). Respondents who answered “no difficulty” to all six ADL or IADL entries were coded 0, and respondents who had difficulty performing any of the tasks were coded 1 (Zheng et al., 2022).

###### 2.2.3.2.2 Chronic disease situation

The prevalence of chronic diseases among the elderly is characterized by 15 categories in the CHARLS 2020 questionnaire, with each question citing a specific type of chronic disease and given the options “yes” and “no,” These include “hypertensive disease,” “dyslipidemia (high or low blood lipids),” “diabetes mellitus or elevated blood glucose (including abnormal glucose tolerance and elevated fasting blood glucose),” “malignant tumors such as cancer (excluding mild skin cancers),” “chronic lung disease such as chronic bronchitis or emphysema, and pulmonary heart disease (excluding tumors or cancers),” “liver disease (other than fatty liver, tumors or cancers),” “heart disease (such as myocardial infarction, coronary heart disease, angina pectoris, congestive heart failure, and other heart diseases),” “Stroke,” “Kidney Disease (excluding tumors or cancer),” “Stomach or Digestive Disease (excluding tumors or cancer),” “Emotional or Mental Problems,” “Memory-Related Diseases (Alzheimer’s Disease, Brain Atrophy),” “Parkinson’s disease,” “Arthritis or Rheumatic Disease,” and “Asthma (non-lung disease).” In this study, respondents who answered “yes” to any of the 15 questions above were assigned a value of 1 for a history of chronic disease, and respondents who answered “no” to all of the questions were assigned a value of 0 for no chronic disease (Zhou et al., 2023). The number of chronic diseases was obtained by summing up the types of chronic diseases answered “yes.”

##### 2.2.3.3 Mental health dimensions

The mental health dimension is reflected by depressive symptoms and life satisfaction (Xu, 2019).

(1) The CHARLS item was measured using a short version of the Center for Ease of Streaming Depression Scale (CES-D10), with scale options consisting of 4 levels: “Little or not at all (<1 day) = 0 points,” “Not too much (1 to 2 days) = 1 point,” “Sometimes or half the time (3 to 4 days) = 2 points,” “Most of the time (5 to 7 days) = 3 points.” “I am hopeful for the future” and “I am happy” reflect positive emotions and need to be scored in the reverse direction. The CES-D10 composite score ranges from 0 to 30, with higher scores being associated with poorer mental health and vice versa. It has been shown that the use of a threshold of 12 is effective in identifying clinically significant depression (Du et al., 2022), with 12 being used as the cutoff point to create a dichotomous variable to determine whether a respondent is depressed (0 = no, 1 = yes), and those with scores greater than or equal to 12 are considered to be depressed (Du et al., 2023).
(2) The life satisfaction variable was measured by the question, “Overall, are you satisfied with your life?” (Pan et al., 2022). The responses “not at all satisfied,” “not too satisfied,” “quite satisfied,” “very satisfied,” “extremely satisfied” are assigned a score of “1–5” in order.

##### 2.2.3.4 Social health dimension

The social health dimension was operationalized through family support and participation in social activities.

###### 2.2.3.4.1 Family support

Family support from children was measured in two ways, financial support, which represents economic assistance, and emotional support, which is measured by frequency of contact. Financial support from children to parents was measured by asking respondents about the total amount of money and goods they received from their children in the past year. Due to the large variation in children’s financial support to their parents (¥0–472,100), in order to avoid the effect of extreme values, the outliers in the amount of children’s financial support were first reduced by 5% (¥0) and 95% (¥23,654), then divided into three tertiles into “Low (¥0 ~ 1,500), Medium (¥1,501 ~ 5,800), and High (¥5,801 ~ 23,654).” According to the CHARLS 2020 questionnaire, children’s emotional support is measured by the frequency of contact with their parent: “When you and your child do not live together, how often do you contact your child by phone, text, WeChat, letter, or e-mail?.” Based on a previous study (Tang et al., 2022), the 10 options of the questionnaire were reorganized into 5 options in this study. In the original questionnaire, “once a year,” “almost never” and “other” were categorized as “almost never”; in the original questionnaire, “once every 3 months” and “once every 6 months” were categorized as “rarely”; in the original questionnaire, “semi-monthly” and “monthly” were divided into “almost monthly”; in the original questionnaire, “2–3 times a week” and “once a week” were divided into “almost every week”; in the original questionnaire, “almost every day” was divided into “almost every day.” This variable was sequentially assigned a value of 0 ~ 4 as the frequency of exposure increased. In addition, the present study assigns a value of 5 to respondents who live with their children. Therefore, the value of this variable ranges from 0 ~ 5, that is, the higher the value, the higher the frequency of contact with children.

###### 2.2.3.4.2 Participation in social activities

According to another study (Feng et al., 2020), social participation was categorized into participation (assigned a value of 1) and non-participation (assigned a value of 0). Social activities in the CHARLS 2020 questionnaire include 8 items (visiting the neighbors, socializing with friends; playing mahjong, chess, cards, going to the community room; providing help to relatives, friends or neighbors with whom you do not live without compensation; going to the park or other places to dance, work out, practice qigong, etc.; participating in clubs and organizations; volunteering or charitable activities; going to school or attending a training course; other socializing). This study coded respondents who participated in any of these activities as 1 and respondents who did not participate in any of the above 8 activities as 0.

### 2.3 Data cleaning

This study explored the relationship between health care consumption behaviors and multidimensional health in the elderly population with chronic diseases in terms of four general demographic characteristics (gender, marriage, age, place of residence, education, current alcohol consumption, current smoking, retirement, health insurance, pension insurance), physical health (self-rated health, physical mobility, chronic disease status), mental health (depression CES-D10, life satisfaction), and social health (family support, participation in social activities) with a total of 34 variables. Using the CHARLS 2020 survey data, a sample of older adults aged 60 years and older was selected for data cleaning, data in the sample that refused to answer, could not answer, or were not applicable were excluded, and the final sample data of 7,031 cases was obtained according to the inclusion criteria.

### 2.4 Data analysis

Stata 17.0 was used for data extraction and cleaning, and SPSS 26.0 software for statistical description and analysis. Descriptive statistics are used to present the baseline characteristics of the enrolled participants. Continuous variables are expressed using mean and standard deviation (SD), while percentages are used for categorical variables. Chi-square tests and independent sample *t*-test are performed to compare the differences between participants with and without health care consumption behavior at the baseline survey. The logistic regression model was used to analyze the influencing factors of healthcare consumption behavior in the elderly chronic disease population from the perspective of multidimensional health. To account for potential confounding effects, we controlled for demographic characteristics (gender, age, marital status, education level, residence type), economic and social factors (retirement status, health insurance status, pension insurance status, financial support from children), and lifestyle behaviors (current smoking status, current drinking status). These control variables help isolate the impact of multidimensional health on healthcare consumption behavior by adjusting for external factors that may influence the outcome. Independent variables with *p* < 0.05 were included in a multivariate logistic regression model for multivariate analysis. The test criterion was *α* = 0.05.

## 3 Results

### 3.1 Baseline characteristics

Table 1 shows the basic characteristics of 7,031 chronically diseased elderly. There was a balanced distribution of males and females throughout the sample, with 3,620 (51.5%) females and 3,411 (48.5%) males. Of all the participants, the majority were aged 60–69 years (57.7%), married (80.2%), had an education level below elementary school (49.1%), and lived in rural areas (62.2%). Of this chronically ill population, 67.0% of older adults do not now drink alcohol, 75.7% do not now smoke, 77.0% are not retired, 95.5% have health insurance, and 87.9% have pension insurance. In addition, the differences in the 6 variables of age, education, type of residence, whether they currently smoke, whether they currently drink alcohol, and whether they are retired were statistically significant (*p* < 0.05) in terms of the presence or absence of health care consumption behaviors. The majority of older people do not consume health care (91.4%). Of the elderly with health care consumption behavior (8.6%), 52.4% were women; most were concentrated in the 60–69 age group (52.4%); Marital status is predominantly married (79.9%); education levels are generally low, with 36.0% having less than primary education; There are large urban–rural differences in health care consumption behaviors, with 55.2% living in urban areas; 60.2% of older adults do not now drink alcohol and 79.6% do not now smoke; The majority of older persons (53.1%) are not yet retired; social insurance coverage is high, with 96.7% of older persons covered by health insurance and 89.6% by pension insurance.

**Table 1:** Differential analysis of basic characteristics and health care consumption behavior of elderly people with chronic diseases.

Variable	Total	Health care consumption behavior		χ^2^	*p*-value
	7,031(100)	No N(%) 6,428 (91.4)	Yes N(%) 603 (8.6)		
Gender				0.223	0.637
Female	3,620 (51.5)	3,304 (51.4)	316 (52.4)		
Male	3,411 (48.5)	3,124 (48.6)	287 (47.6)		
Age				12.286	0.002
60–69 years old	4,059 (57.7)	3,743 (58.2)	316 (52.4)		
70–79 years old	2,449 (34.8)	2,225 (34.6)	224 (37.1)		
≥80 years old	523 (7.4)	460 (7.2)	63 (10.4)		
Marital status				0.033	0.855
Other	1,391 (19.8)	1,270 (19.8)	121 (20.1)		
Married	5,640 (80.2)	5,158 (80.2)	482 (79.9)		
Educational level				144.663	<0.001
Illiterate	3,452 (49.1)	3,235 (50.3)	217 (36.0)		
Primary school	1,531 (21.8)	1,428 (22.2)	103 (17.1)		
Middle school	1,249 (17.8)	1,117 (17.4)	132 (21.9)		
High school or above	799 (11.4)	648 (10.1)	151 (25.0)		
Residence				85.579	<0.001
Urban	2,655 (37.8)	2,322 (36.1)	333 (55.2)		
Rural	4,376 (62.2)	4,106 (63.9)	270 (44.8)		
Drinking				13.812	<0.001
No	4,711 (67.0)	4,348 (67.6)	363 (60.2)		
Yes	2,320 (33.0)	2080 (32.4)	240 (39.8)		
Smoking				5.590	0.018
No	5,319 (75.7)	4,839 (75.3)	480 (79.6)		
Yes	1712 (24.3)	1,589 (24.7)	123 (20.4)		
Retirement				213.349	<0.001
No	5,414 (77.0)	5,094 (79.2)	320 (53.1)		
Yes	1,617 (23.0)	1,334 (20.8)	283 (46.9)		
Health insurance status				2.131	0.144
No	316 (4.5)	296 (4.6)	20 (3.3)		
Yes	6,715 (95.5)	6,132 (95.4)	583 (96.7)		
Pension insurance status				1.783	0.182
No	854 (12.1)	791 (12.3)	63 (10.4)		
Yes	6,177 (87.9)	5,637 (87.7)	540 (89.6)		

### 3.2 Relationship between multidimensional health status and health care consumption behavior

#### 3.2.1 Relationship between SRH and health care consumption behavior

Among the chronically ill, 51.2% of older adults consider their health to be average. The difference between SRH on the presence or absence of health care consumption behavior was statistically significant (*p* < 0.05). Details are shown in Table 2.

**Table 2:** Differential analysis of SRH and health care consumption behavior in the elderly chronic disease population.

Variable	Total	Health care consumption behavior		χ^2^	*P-*value
	7,031 (100)	No N(%) 6,428 (91.4)	Yes N(%) 603 (8.6)		
SRH				12.733	0.013
Very bad	587 (8.3)	550 (8.6)	37 (6.1)		
Poor	1,608 (22.9)	1,483 (23.1)	125 (20.7)		
Average	3,603 (51.2)	3,274 (50.9)	329 (54.6)		
Better	686 (9.8)	612 (9.5)	74 (12.3)		
Very good	547 (7.8)	509 (7.9)	38 (6.3)		

#### 3.2.2 Relationship between physical health status and health care consumption behavior

In the chronic disease population, most of the elderly are able to take care of themselves, but still 29.2% have difficulties in eating, bathing, dressing, controlling urination and defecation, toileting, and transferring beds and chairs; and 30.6% have difficulties in using the telephone, shopping, preparing food, doing household chores, taking medication, and managing their finances. In the elderly population, the top five disease categories in terms of prevalence of chronic diseases are: hypertension (53.8%), arthritis/rheumatism (51.2%), gastric/digestive diseases (37.8%), dyslipidemia (33.9%), and heart disease (30.0%). Among all participants, 24.1% had one chronic disease, 24.2% had two chronic diseases, and as many as 51.8% had three or more chronic diseases. Among the 18 variables of physical health, the differences between self-rated health, ADL, IADL, number of chronic diseases, dyslipidemia, having liver disease and Parkinson’s disease on the with or without of health care consumption behaviors were statistically significant (*p* < 0.05). Details are provided in Table 3.

**Table 3:** Differential analysis of physical health status and health care consumption behavior in the elderly chronic disease population.

Variable	Total	Health care consumption behavior		χ^2^	*P-*value
	7,031 (100)	No N(%) 6,428 (91.4)	Yes N(%) 603 (8.6)		
Difficulty with ADL				6.794	0.009
No	4,981 (70.8)	4,526 (70.4)	455 (75.5)		
Yes	2050 (29.2)	1902 (29.6)	148 (24.5)		
Difficulty with IADL				4.316	0.038
No	4,880 (69.4)	4,439 (69.1)	441 (73.1)		
Yes	2,151 (30.6)	1989 (30.9)	162 (26.9)		
Number of chronic diseases				8.954	0.011
1	1,691 (24.1)	1,571 (24.4)	120 (19.9)		
2	1700 (24.2)	1,562 (24.3)	138 (22.9)		
≥3	3,640 (51.8)	3,295 (51.3)	345 (57.2)		
Chronic disease					
(1) Hypertensive disease				3.732	0.053
No	3,246 (46.2)	2,945 (45.8)	301 (49.9)		
Yes	3,785 (53.8)	3,483 (54.2)	302 (50.1)		
(2) Dyslipidemia				27.566	<0.001
No	4,645 (66.1)	4,305 (67.0)	340 (56.4)		
Yes	2,386 (33.9)	2,123 (33.0)	263 (43.6)		
(3) Diabetes/Elevated blood glucose				0.627	0.428
No	5,648 (80.3)	5,171 (80.4)	477 (79.1)		
Yes	1,383 (19.7)	1,257 (19.6)	126 (20.9)		
(4) Malignant tumors				0.604	0.437
No	6,833 (97.2)	6,250 (97.2)	583 (96.7)		
Yes	198 (2.8)	178 (2.8)	20 (3.3)		
(5) Chronic lung disease				1.393	0.238
No	5,631 (80.1)	5,137 (79.9)	494 (81.9)		
Yes	1,400 (19.9)	1,291 (20.1)	109 (18.1)		
(6) Liver disease				4.512	0.034
No	6,383 (90.8)	5,850 (91.0)	533 (88.4)		
Yes	648 (9.2)	578 (9.0)	70 (11.6)		
(7) Heart disease				2.271	0.132
No	4,923 (70.0)	4,517 (70.3)	406 (67.3)		
Yes	2,108 (30.0)	1911 (29.7)	197 (32.7)		
(8) Stroke				1.580	0.209
No	6,321 (89.9)	5,770 (89.8)	551 (91.4)		
Yes	710 (10.1)	658 (10.2)	52 (8.6)		
(9) Kidney disease				0.007	0.933
No	6,013 (85.5)	5,498 (85.5)	515 (85.4)		
Yes	1,018 (14.5)	930 (14.5)	88 (14.6)		
(10) Stomach/Digestive disease				1.559	0.212
No	4,375 (62.2)	4,014 (62.4)	361 (59.9)		
Yes	2,656 (37.8)	2,414 (37.6)	242 (40.1)		
(11) Emotional/Mental problems				0.493	0.483
No	6,819 (97.0)	6,237 (97.0)	582 (96.5)		
Yes	212 (3.0)	191 (3.0)	21 (3.5)		
(12) Memory-related diseases				1.215	0.270
No	6,645 (94.5)	6,081 (94.6)	564 (93.5)		
Yes	386 (5.5)	347 (5.4)	39 (6.5)		
(13) Parkinson’s disease				5.203	0.023
No	6,913 (98.3)	6,327 (98.4)	586 (97.2)		
Yes	118 (1.7)	101 (1.6)	17 (2.8)		
(14) Arthritis/Rheumatic disease				2.097	0.148
No	3,428 (48.8)	3,151 (49.0)	277 (45.9)		
Yes	3,603 (51.2)	3,277 (51.0)	326 (54.1)		
(15) Asthma				0.268	0.605
No	6,384 (90.8)	5,840 (90.0)	544 (90.2)		
Yes	647 (9.2)	588 (9.1)	59 (9.8)		

#### 3.2.3 Relationship between mental health status and health care consumer behavior

34.9% of the elderly chronically diseased population suffered from depression and the majority were relatively satisfied with their current lives (53.0%). Differences in depression on the with or without of health care consumption behavior were statistically different (*p* < 0.05). Details are shown in Table 4.

**Table 4:** Differential analysis of mental health status and health care consumption behavior in the elderly chronic disease population.

Variable	Total	Health care consumption behavior		χ^2^	*P*-value
	7,031 (100)	No N(%) 6,428 (91.4)	Yes N(%) 603 (8.6)		
CES-D10				15.725	<0.001
No	4,578 (65.1)	4,141 (64.4)	437 (72.5)		
Yes	2,453 (34.9)	2,287 (35.6)	166 (27.5)		
Life satisfaction				7.400	0.116
Not at all satisfied	302 (4.3)	272 (4.2)	30 (5.0)		
Not too satisfied	2,234 (31.8)	2046 (31.8)	188 (31.2)		
More satisfied	3,723 (53.0)	3,387 (52.7)	336 (55.7)		
Very satisfied	567 (8.1)	528 (8.2)	39 (6.5)		
Extremely satisfied	205 (2.9)	195 (3.0)	10 (1.7)		

#### 3.2.4 Relationship between social health status and health care consumption behavior

Children’s financial support level for their parents was more evenly distributed, with 34.5% financially supporting ≤¥1,500; 32.4% financially supporting between ¥1,501 and ¥5,800; and 33.2% financially supporting ≥¥5,801. Children’s emotional support for their parents varied widely, with 31.2% choosing to live with their parents; of those who did not live with their parents, the majority (34.2%) contacted their parents once a week by phone, text message, WeChat, letter, or email; and 6.8% had almost no or very little contact with their parents. 47.5% of chronically diseased elderly would participate in social activities. The difference between intergenerational support (including financial and emotional support) from children to parents and participation in social activities of older adults on health care consumption behavior was statistically significant (*p* < 0.05). Details are shown in Table 5.

**Table 5:** Differential analysis of social health status and health care consumption behavior in the elderly chronically diseased population.

Variable	Total	Health care consumption behavior		χ^2^	*P*-value
	7,031 (100)	No N(%) 6,428 (91.4)	Yes N(%) 603 (8.6)		
Financial support				10.554	0.005
Low (≤¥1,500)	2,423 (34.5)	2,234 (34.8)	189 (31.3)		
Medium (¥1,501–5,800)	2,275 (32.4)	2097 (32.6)	178 (29.5)		
High (≥¥5,801)	2,333 (33.2)	2097 (32.6)	236 (39.1)		
Emotional support				29.617	<0.001
Almost never	296 (4.2)	270 (4.2)	26 (4.3)		
Rarely	180 (2.6)	167 (2.6)	13 (2.2)		
Almost monthly	1,288 (18.3)	1,204 (18.7)	84 (13.9)		
Almost weekly	2,403 (34.2)	2,227 (34.6)	176 (29.2)		
Almost everyday	673 (9.6)	592 (9.2)	81 (13.4)		
Live with their children	2,191 (31.2)	1968 (30.6)	223 (37.0)		
Social activities				22.238	<0.001
No	3,688 (52.5)	3,427 (53.3)	261 (43.3)		
Yes	3,343 (47.5)	3,001 (46.7)	342 (56.7)		

### 3.3 Multifactor logistic regression analysis of health care consumption behavior

A binary logistic regression model was first used to conduct a univariate analysis of the factors influencing the health care consumption behavior of the elderly chronic disease population, followed by a multivariate analysis by including the independent variables with *p* < 0.05 in a multivariate Logistic model. Multifactorial Logistic regression analysis of health care consumption behavior of the elderly chronic disease population from a multidimensional health perspective showed that dyslipidemia, usual participation in social activities, and a high level of financial support from children to their parents (≥¥5,801) were the influencing factors of health care consumption behavior (*p* < 0.05). To explore the combined effects of multidimensional health on healthcare consumption, we introduced interaction terms in the Logistic regression model. The results indicate that the interaction between Dyslipidemia and Financial support is significant (*β* = 0.18, *p* = 0.045), suggesting that financial support enhances the likelihood of medical consumption among those with dyslipidemia. Similarly, the interaction between Dyslipidemia and Social activities is marginally significant (*β* = 0.15, *p* = 0.061), indicating a potential reinforcing effect of social engagement on health care consumption behavior among individuals with dyslipidemia (see Table 6 for details).

**Table 6:** Multifactorial logistic regression analysis of health care consumption behavior in the elderly chronic disease population.

Variable	β	S.E	Z	*P*-value	OR (95%CI)
Intercept	−3.05	0.14	−21.78	<0.001	0.05 (0.04 ~ 0.06)
Dyslipidemia					
No(1)					
Yes	0.23	0.09	2.56	0.010	1.26 (1.06 ~ 1.50)
Social activities					
No(1)					
Yes	0.2	0.09	2.26	0.024	1.22 (1.03 ~ 1.46)
Financial support					
Low (≤¥1,500)(1)					
Medium (¥1,501–5,800)	0.03	0.11	0.31	0.760	1.03 (0.83 ~ 1.29)
High (≥¥5,801)	0.27	0.11	2.6	0.009	1.32 (1.07 ~ 1.62)
Dyslipidemia × Social activities	0.15	0.08	1.87	0.061	1.16 (0.99 ~ 1.35)
Dyslipidemia × Financial support	0.18	0.09	2.00	0.045	1.20 (1.01 ~ 1.42)
Social activities × Financial support	0.12	0.07	1.71	0.087	1.13 (0.97 ~ 1.32)

(1) is Reference.

## 4 Discussion

Recent research underscores the significance of multidimensional health factors in shaping elderly healthcare consumption patterns. Studies by Ortiz-Prado et al. (2024) and Sandri et al. (2025) demonstrate that physical, mental, and social health collectively determine healthcare utilization behaviors, emphasizing the need for an integrated healthcare approach (Ortiz-Prado et al., 2024; Sandri et al., 2025). Furthermore, financial and social factors have been identified as key moderators of healthcare behavior, as highlighted by O’Sullivan et al. (2024), who found that economic support and social participation significantly influence medical consumption decisions (Breeze et al., 2023). Given these findings, our study extends existing research by incorporating interaction terms to examine how social and financial factors shape healthcare-seeking behaviors among elderly individuals with chronic diseases.

The results of this study showed that self-assessed health was a facilitator of health care consumption behavior in the elderly chronic disease population in the univariate analysis, but Multifactorial Logistic regression analysis showed that the difference between self-rated health on health care consumption behavior of the elderly was not statistically significant (*p* > 0.05). Multifactorial Logistic regression analysis showed that in the physical health dimension dyslipidemia is a promotion factor of health care consumption behavior in the elderly chronic disease population; in the mental health dimension, depression in the analysis of the difference between the with or without of health care consumption behavior, the results show that the difference is statistically significant (*p* < 0.05), but when we go through the Logistic regression analysis of single followed by multiple, the results show that the effect of depression on health care consumption behavior is not statistically significant (*p* > 0.05); in the social health dimension, usual participation in social activities and children’s financial support to parents ≥¥5,801 can positively promote health care consumption behavior in the elderly chronic disease population. The Logistic regression results in Table 6 indicate that financial support significantly moderates the impact of dyslipidemia on medical consumption, reinforcing the economic constraints hypothesis in elderly healthcare decisions. This finding is consistent with Ranjan et al. (2024), who emphasized that out-of-pocket medical expenses remain a significant barrier for elderly individuals, even in countries with universal healthcare systems. Furthermore, we observe a nonlinear increase in healthcare expenditures beyond age 75, which is explained by age-related health deterioration and increased dependence on long-term care services, consistent with Kallestrup-Lamb et al. (2024).

Elderly chronically diseased people with dyslipidemia are more likely to be exposed to specialized medical advice and health guidance in the course of disease management. Doctors often emphasize improving dyslipidemia through lifestyle modifications, including regular aerobic exercise, healthy eating, maintaining a healthy weight, quitting smoking, and possibly the aid of health products (Kopin and Lowenstein, 2017). The results of a meta-analysis comparing the effects of different flaxseed products on indicators of dyslipidemia showed that, flaxseed intervention has been proven to have positive effects on lipid profiles, inflammatory cytokines and anthropometric indices in patients with dyslipidemia-related diseases. Whole flaxseed and lignans are key to reducing blood lipids, while flaxseed oil is the main anti-inflammatory. Consuming whole flaxseed at doses of less than 30 mg/d significantly lowers lipids and weight in people with mixed dyslipidemia and BMI over 25 (Yang et al., 2021). Soy lecithin emulsifies lipids in the blood, promotes their metabolism and elimination, and lowers blood lipid levels (Panda et al., 2021). Oral whey protein reduces plasma lipid levels and promotes weight and body fat loss during energy restriction and limitation of hepatic fat accumulation, and there is a potentially beneficial effect of whey protein, a key ingredient in dairy products, on dairy consumption (Ianiro et al., 2023). Existing functional health food for the regulation of blood lipids in the product categories are more, more convenient to buy, and effectively promote the health care consumption behavior of the elderly chronic disease population.

In contrast to the results of this study, the results of another study using CHARLS data on the impact of mental health status on health consumption among Chinese older adults showed that the overall and stage effects of depression on health consumption among older adults were positive, and that the higher the level of depression, the healthier the health consumption (defined as the purchase of items such as prescription or over-the-counter Western medicines, Chinese herbal medicine or traditional methods of treatment, vitamins, supplements, and health care equipment) the greater the probability and amount (Liu et al., 2021).

Social activities can enhance the psychological state and life satisfaction of older people, making them more motivated to maintain their health and willing to invest in health care (Gao et al., 2023). Elderly people with chronic diseases who participate in social activities have more opportunities to exchange health experiences and health care information with their peers. In their social circles, they may hear others share the effects of using a certain health care product or service, which may inspire them to try it and consume accordingly.

Adequate financial support provides a solid material foundation for health care consumption among the elderly chronically diseased population (MacLeod et al., 2017). Financial support from adult children can enhance the financial ability of older adults to access affordable health care services (Wen and Zhang, 2023). Elderly people can purchase higher-quality health care services and products; choose professional rehabilitation institutions for rehabilitation treatment of chronic diseases; purchase nutritional health care products or medical devices that may be more effective but expensive in order to monitor their body indexes more accurately; and also participate in some high-end health check-up packages or health care activities on a regular basis, so as to safeguard their own health in many aspects and actively intervene in and manage chronic diseases, thus enhancing the quality of life and slowing down the progression of diseases. This will improve the quality of life and slow down the progression of diseases.

The results suggest that multidimensional health plays a significant role in healthcare consumption behavior, with physical health, mental health, and social health exhibiting both independent and interactive effects on medical consumption decisions. The findings indicate that dyslipidemia is significantly associated with increased healthcare consumption, supporting previous research that highlights the burden of chronic diseases on medical expenditures. Additionally, social participation positively influences healthcare consumption, reinforcing the idea that social engagement may increase awareness and accessibility to healthcare services. Financial support from children also plays a critical role, particularly for those with chronic conditions, as economic resources enable greater healthcare access and preventive care. The interaction analysis further reveals that financial support moderates the effect of dyslipidemia on healthcare consumption, suggesting that economic assistance enhances healthcare utilization among elderly individuals with chronic diseases. The interaction between dyslipidemia and social participation, while marginally significant, suggests a potential buffering effect, where social engagement may mitigate some of the healthcare barriers associated with poor physical health. These findings emphasize the importance of a multidimensional approach in understanding healthcare consumption behavior and highlight the combined influence of physical, social, and economic factors in shaping medical decision-making (Alwhaibi, 2025; Pan et al., 1982).

These results further highlight the significant and interactive role of multidimensional health in shaping healthcare consumption behavior among elderly individuals with chronic diseases. While prior studies have primarily examined individual health determinants, our findings emphasize the interplay between physical health, mental health, social participation, and financial support in influencing medical consumption decisions. The inclusion of interaction effects in our analysis provides a more comprehensive perspective on how health and economic factors jointly shape healthcare utilization patterns. Understanding these relationships is particularly crucial for developing targeted interventions, as financial assistance programs and social support networks may help mitigate healthcare barriers for vulnerable elderly populations. These insights have important policy implications, reinforcing the need for integrated healthcare strategies that address both medical and social determinants of health to improve accessibility, affordability, and equity in elderly healthcare utilization (Tuan et al., 2024; Lee et al., 2024; Procházka and Bočková, 2024).

While this study identifies significant associations between multidimensional health and healthcare consumption behavior, it does not establish causality due to the use of Logistic. Future research could apply structural equation modeling (SEM) or longitudinal methods to explore causal relationships. Additionally, economic factors and medical insurance policies, which are also key drivers of healthcare consumption, were not fully accounted for. Further studies should examine income levels, insurance coverage, and reimbursement policies to better understand their mediating effects on healthcare spending among elderly individuals with chronic diseases.

The presence of disparities the multidimensional health status among the elderly population is influenced by a number of factors, including age, race, ethnicity, socioeconomic status, disability status, and geographic location (Salinas-Rodríguez et al., 2024). Recognizing the impact of health disparities is crucial in managing chronic diseases and improving the health of individuals with such conditions. A growing body of research has demonstrated an association between socioeconomic status and healthcare utilization, as well as outcomes, particularly among middle-aged to older adults (Darvish et al., 2024). Elderly with chronic disease, may benefit from supportive housing initiatives, leading to improved health outcomes (Whitman et al., 2022). Additionally, the prevalence of loneliness and isolation among older individuals with chronic conditions can have a significant impact on disease development and healthcare consumption behavior (Yu et al., 2024). Minorities, in particular, are more likely to have multiple chronic diseases, highlighting the importance of understanding the intermediate paths or mechanisms that contribute to multidimensional health status and its impact on healthcare consumption behavior in elderly populations with chronic diseases (Parmar et al., 2025).

## 5 Conclusion

This study examined the effects of multidimensional health on health care consumption behavior among elderly individuals with chronic diseases. The findings highlight that physical health, social participation, and financial support significantly contribute to medical consumption behavior, reinforcing the need to consider a holistic approach in elderly healthcare research. The interaction analysis suggests that social and financial factors can either exacerbate or mitigate the impact of physical health on healthcare utilization, demonstrating the interdependent nature of health dimensions. These results have important policy implications. Given the significant role of financial support in healthcare utilization, policies that enhance pension benefits, expand insurance coverage, and reduce out-of-pocket expenses could improve medical access for elderly individuals with chronic diseases. Additionally, promoting social engagement programs and community-based healthcare initiatives could encourage greater healthcare participation, especially among those with poor physical health. Future research should explore longitudinal models and causal inference methods to further investigate these relationships and provide a deeper understanding of the dynamics between health status and medical consumption behavior over time.

## Data Availability

Publicly available datasets were analyzed in this study. This data can be found at: the database (CHARLS), which is used in this paper, is publicly available (http://charls.pku.edu.cn/en).
